# Induction of an immune response by a nonreplicating adenoviruses-based formulation versus a commercial pseudo-SARS-CoV-2 vaccine

**DOI:** 10.5114/bta.2024.141805

**Published:** 2024-09-30

**Authors:** Joanna Baran, Łukasz Kuryk, Mariangela Garofalo, Katarzyna Pancer, Magdalena Wieczorek, Michalina Kazek, Monika Staniszewska

**Affiliations:** 1 Centre for Advanced Materials and Technologies, Warsaw University of Technology, Warszawa, Poland; 2 National Institute of Public Health National Institute of Hygiene (NIH) – National Institute of Research, Warszawa, Poland; 3 Department of Pharmaceutical and Pharmacological Sciences, University of Padova, Padova, Italy

**Keywords:** immune response, adenoviruses, vaccines, SARS-CoV-2, CD cells

## Abstract

Screening for effective vaccines requires broad studies on their immunogenicity *in vitro* and *ex vivo* . We used a PBMC-based system to assess changes in CD4^+^ T cells, CD8^+^ T cells, and CD19^+^ B cells upon stimulation with different combinations of antigens and adjuvants. We studied the activation mechanism using flow cytometry and two different adenoviral adjuvants characterized by the presence or absence of costimulatory ligands for the ICOS and CD40 receptors. Our studies identified the cellular targets and molecular mechanisms driving ongoing switched-antibody diversification. Class-switched memory B cells were the main precursor cells (95.03% ± 0.38 vs. mock 82.33% ± 0.45, *P* < 0.05) after treatment with the immunogenic formula: adenovirus armed (MIX1) or not (MIX2) with the ICOS and CD40 ligand, the recombinant receptor binding domain (rRBD), and Lentifect™ SARS-CoV-2 spike-pseudotyped lentivirus (GeneCopoeia, USA). Bcell class-switching towards the IgG^+^IgM^+^- positive phenotypes was noted (~50-fold increase vs. mock, *P* < 0.05). A significant increase was observed in the CD8^+^T_EM_ population of the MIX1 (~2-fold, *P* < 0.05) and MIX2 (~4.7-fold, *P* < 0.05) treated samples. CD8^+^T_EMRA_ increased when PBMCs were treated with MIX2 (9.63% ± 0.90, *P* < 0.05) vs. mock (2.63% ± 1.96). Class-switched memory B cells were the dominant antigen-specific cells in primary reactions. We indicated a correlation between the protection offered by vaccine regimens and their ability to induce high frequencies of multifunctional T cells.

## Introduction

Infectious diseases cause many health and economic hazards. As travel becomes more popular, infectious diseases have more pathways to spread, posing a risk to the local population and a global epidemic threat (Excler et al., 2021; Wang et al., 2021). An example is the coronavirus disease 2019 (COVID-19), which spread dynamically, causing a pandemic. The development of new, rapid pathways for screening therapeutic approaches should be a priority.

In this publication, we propose a system based on peripheral blood mononuclear cells (PBMCs) isolated from healthy donors for *in vitro* immunogenicity testing of vaccine formulation ingredients. PBMCs contain different blood cells, including monocytes, lymphocytes, and macrophages (Kleiveland, 2015). They have proven to be a valuable immune response model in the studies of immunotherapeutics (Tapia-Calle et al., 2019).

Proprietary formulations based on nonreplicating adenoviruses (AdVs) Ad5/3-D24-ICOSL-CD40L (AdV1) and Ad5/3-D24-WT (AdV2) were used for the research (Garofalo et al., 2021a). We reported using the AdVs as promising vaccine adjuvants (Garofalo et al., 2020). Enhancement of the immunogenicity of the vaccine antigens and boosting the development of a proinflammatory response were demonstrated. It is worth emphasizing that AdVs have been tested in numerous preclinical and clinical studies, determining their safety in various therapies (Ranki et al., 2014, 2016; Vassilev et al., 2015). AdV1 and AdV2 have the chimeric serotype 5/3, in which the fiber knob five domain has been replaced with the fiber knob three domain and a 24 bp deletion in the *E1A Conserved Region 2* region. This modification ensures no replication in healthy cells. AdV1 encodes two costimulatory ligands: ICOSL (inducible costimulator ligand) and CD40L. It is well known that costimulatory ligands are crucial for T cell activation, leading to the enhancement of the immune response and the extension of cell memory (Croft, 2003). ICOSL interacts with the ICOS molecule in activated T lymphocytes, enhancing the T cell response to a foreign antigen. This process promotes the guidance of immune cells into inflamed tissue (Garofalo et al., 2021a).

The recombinant receptor binding domain (rRBD) and the commercial lentivirus pseudotyped with spike protein were used to study immunogenesis and vaccine design. We tested how the combinations of adjuvants and antigens influence the CD8^+^ and CD4^+^ T cell response. The humoral response was expressed by phenotyping B lymphocytes (CD19^+^). The major questions were: 1) How does the vaccine immune-stimulation induce the PBMC response? 2) What influence do the proposed formulations have on the expression of the CD40 gene (under/over-expression)? The results were supported by flow cytometric and RT-qPCR analysis.

## Materials and methods

### Viruses, media, and rRBD

The VERO E6 cell line ATCC (LGC Standard, Lomianki, Poland) was used as an infection model (Ogando et al., 2020). The VERO E6 cells were cultured in Minimum Essential Media (MEM) ATCC (LGC Standard, Lomianki, Poland), supplemented with 1% penicillin/streptomycin (Gibco Laboratories, USA), 5 mM sodium pyruvate (Gibco Laboratories, USA), and 10% fetal bovine serum (FBS, Gibco Laboratories, USA). The following adenoviruses were used: AdV-D24-ICOSL-CD40L (5.2×10^11^ VP/ml), named AdV1, and AdV5.3-d24-E3 (7.7×10^12^ VP/ml), named AdV2 (Garofalo et al., 2021b). The Lentifect™ SARS-CoV-2 Spike-pseudotyped lentivirus was obtained from GeneCopoeia (1 × 10^7^ RLU/ml).

The expression of the rRBD gene was described by Baran et al. (2023). Briefly, genetic engineering methods were used to construct the rRBD gene in prokaryotic expression vectors. The RBD gene sequence used for protein expression was designed using the amino acid sequence. The nucleotide sequence of the gene was optimized for bacterial codon usage. Primers and synthetic oligonucleotides were designed and used for the construction of the gene with a tag at the N-terminus. The prokaryotic expression vector including the RBD gene was transformed into an *Escherichia coli* expression strain. The tag at the N-terminus allowed the use of a simple affinity chromatography method for protein purification, which, together with high efficiency in protein expression, significantly reduced manufacturing costs. Appropriate culture media and conditions were experimentally selected for recombinant protein laboratoryscale production. At this stage, a method for isolating and purifying the recombinant protein using medium-pressure affinity chromatography was developed.

### PBMC isolation and processing

PBMCs were isolated from buffy coats obtained from healthy donors, and purchased from the Regional Centre for Blood and Blood Treatment in Warsaw, Poland. Ficoll-Hypaque density gradient centrifugation was used for isolation. Briefly, fresh buffy coats were diluted 1 : 1 with Roswell Park Memorial Institute 1640 medium (RPMI-1640, Gibco Laboratories, USA). Then, 20 ml of Ficoll-Hypaque was transferred to a fresh 50 ml Falcon tube and gently overlaid with the diluted buffy coat to a total volume of 45 ml. The samples were centrifuged at 760×*g* for 20 min with the brakes off (Centrifuge 5910 R, Eppendorf). The PBMC layer was collected and transferred to fresh Falcon tubes. Three washes with RPMI-1640 were performed, after which the cells were centrifuged at 350 ×*g* for 8 min with brakes on. PBMCs were cryopreserved in a freezing medium containing 50% FBS and 20% DMSO (1#x00B0;C/min) and stored for testing in a low-temperature freezer at −150#x00B0;C (MDF-C2156VAN-PE, PHCBI).

For the tests, PBMCs were thawed, washed with fresh OptiMEM medium (Gibco Laboratories, USA), and seeded in 24-well plates at a concentration of 5 × 10^6^ cells/well in OptiMEM supplemented with 10% FCS. After 16–18 h of rest, the cell growth medium was replaced, and the cells were treated with 1.25 μg/ml of LPS (eBioscience^™^, Invitrogen^™^) to stimulate proliferation. PBMCs in 24-well plates were treated with the following immunogenic factors: AdV-D24-ICOSL-CD40L (AdV1; 100 VP/ml; stock 3.2 × 10^3^ TCID_50_/ml), AdV-D24-WT (AdV2; 100 VP/ml; stock 3.2 × 10^6^ TCID_50_/ml), rRBD (2.62 μg/ml), pseudo-SARS-CoV-2 (100 VP/ml; Lentifect ™ SARS-CoV-2 Spike-pseudotyped lentivirus), and SARS-CoV-2 Spike Antibody (GeneCopoeia; 2.62 μg/ml), and incubated for 24 h. Each component was tested in the presence of LPS (1.25 μg/ml). Additionally, PBMCs were treated with the following mixtures of factors: AdV5/3-d24-ICOSL-CD40L+ rRBD+ pseudo-SARS-CoV-2 (100 VP/ml + 2.62 μg/ml + 100 VP/ml), named MIX1, and AdV5/3-d24-WT + rRBD + pseudo-SARS-CoV-2 (100 VP/ml + 2.62 μg/ml + 100 VP/ml), named MIX2.

### Flow cytometry FACS Lyric flow cytometer (BD Biosciences NJ, USA)

A FACS Lyric flow cytometer (BD Biosciences, NJ, USA) was used to study the humoral response (CD19^+^). PBMCs (10^6^/ml) were freshly thawed and rested overnight. Then, the cells were stimulated with the immunogenic factors for 24 h at 37#x00B0;C in 5% CO_2_. The immunophenotyping of the PBMCs was described by Baran et al. (2023). Briefly, the cells were collected, washed, and centrifuged. The cell pellet was suspended in the staining buffer and incubated for 30 min at RT (protected from light). PBMCs stained with CD19 BB700 (cat. No. 566396, BD USA), CD27 APC (cat. no. 561297, BD USA), CD38 PE (cat. no. 555460, BD USA), IgM BB515 (cat. no. 56422, BD USA), IgD (cat. no. PE-Cy7, BD USA), and IgG APC-H7 (cat. no. 561297, BD USA) were used in the experiments. The measurement was assessed with a FACS Lyric flow cytometer (BD Biosciences, NJ, USA).

### Reverse transcription-quantitative real-time polymerase chain reaction (RT-qPCR)

RT-qPCR was conducted as explained in the article by Baran et al. (2023). Briefly, RNA was isolated from VERO E6 cells using the Total RNA Mini Kit (A&A Biotechnology, Gdansk, Poland) and reverse-transcribed using the High-Capacity cDNA Reverse Transcription Kit with RNase Inhibitor (ThermoFisher Scientific, Waltham, USA). cDNA was diluted to a similar concentration to a housekeeping gene (GAPDH), and 1 μl was added to 20 μl of PCR reactions containing random primers, MultiScribe™ Reverse Transcriptase, and buffer (ThermoFisher Scientific, Waltham, USA, Cat. 4374966). Real-time PCR was performed on a CFX96 (Bio-Rad, USA) for 5 min at 95°C, followed by 45 cycles of 95°C for 30 s, 61#x00B0;C for 1 min, 72°C for 1 min, and melting at 50–95°C. The CD40 gene expression was measured, and the GAPDH reference gene was used to normalize target gene levels relative to mRNA levels. The sequences of the primers used were published previously in Baran et al. (2023) (Table S1 and Fig. S1).

### Confocal laser scanning microscopy (CLSM; Zeiss, Germany)

The pseudo-SARS-CoV-2 internalization was checked using CLSM (Zeiss, Germany). A Vero E6 cell suspension at a density of 1 × 10^6^ cells/ml of MEM (10% FBS, 1% Penicillin/Streptomycin, 1% sodium pyruvate) was added to the tested wells. After incubation at 37°C for 24 h with 5% CO_2_, the medium was replaced with a fresh portion of MEM. The cells were incubated for 24 h with either pseudo-SARS-CoV-2 alone (10^-6^ RFU/ml) or with a pseudo-SARS-CoV-2+antibody cocktail (> 1000 RFU/ml + 5.24 μg/ml). The nucleus was stained with propidium iodide (PI).

### Statistical analysis

The statistical analysis of quantitative and qualitative data was performed. Continuous data are expressed as mean± SD unless otherwise specified. Tests to evaluate multiple variables were also performed, including the unpaired *t*-test, one-way ANOVA, two-way ANOVA, and one-sample Wilcoxon test. All statistical tests with *P*-values ≤ 0.05 were considered significant. Data were analyzed with Statistica (StatSoft Inc).

## Results

### Mixture of adenovirus adjuvant, rRBD antigen, and pseudo-SARS-CoV-2 (MIX1) causes an increase in the CD19^+^ B cell responses

**Fig. 1:**
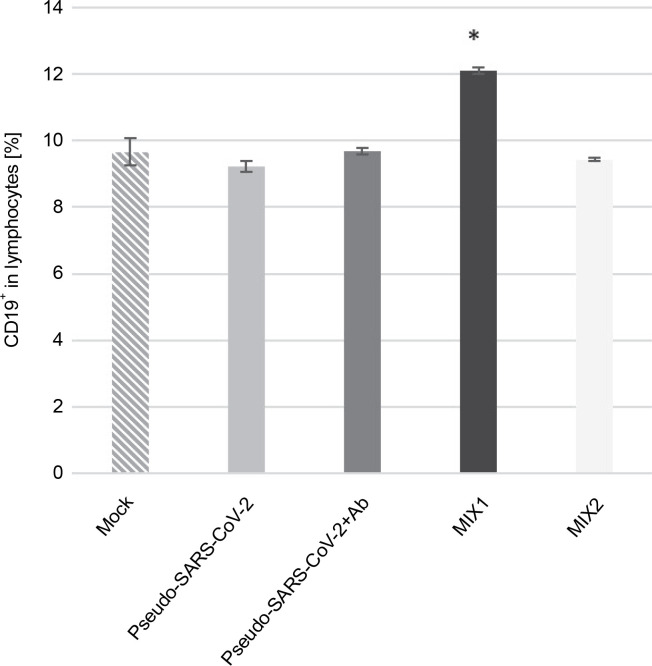
Frequency of CD19^+^ (%) exposed to the immunogenic factors; PBMCs were treated with the following immunogenic factors: Mock – LPS-treated cells (1.25 μg/ml), Pseudo-SARS-CoV-2 – GeneCopoeia’s Lentifect™ SARS-CoV-2 spike-pseudotyped lentivirus (100 VP/ml), Pseudo-SARS-CoV-2 +Ab – pseudo-SARS-CoV-2 + SARS-CoV-2 spike antibody (GeneCopoeia; 100 VP/ml + 2.62 μg/ml), MIX1 – AdV5/3-d24-ICOSL-CD40L + rRBD+ pseudo-SARS-CoV-2 (100 VP/ml + 2.62 μg/ml + 100 VP/ml), MIX2 – AdV5/3-d24-WT + rRBD+ pseudo-SARS-CoV-2 (100 VP/ml + 2.62 μg/ml + 100 VP/ml); the acquisition was performed 24 h postexposition to immunogens using a flow cytometer (FACS Lyric, BD Bioscience, NJ, USA); data are representative of three independent experiments; *P-value ≤ 0.05

xMIX1 resulted in a ~25% increase in CD19^+^ cells compared to the mock (*P* ≤ 0.05; Fig. 1). Contrariwise, based on analyses with the same set of markers, pseudo-SARS-CoV-2 without antibodies or MIX2 generated a similar number of CD19+ cells compared to the mock. We concluded that the emergence of CD19^+^ cells was dependent on the presence of the adjuvant (AdV1) armed with the CD40 ligand. Moreover, we used flow cytometry to analyze the CD19^+^ B cell subset changes in pooled lymphocytes (Table 1). Among the B cells, the percentage of memory B cells was 95.03%±0.38 compared to 82.33% ± 0.45 in the mock. We observed a ~2.6-fold higher percentage of CD38^-^CD24^+^CD27^+^ cells compared to the mock (*P* ≤ 0.05).

**Table 1:** Percentage of the CD19^+^ subpopulations measured by flow cytometry (FACS Lyric, BD Biosciences, NJ, USA)

CD19^+^ subpopulations	Immunogenic factors				
	Mock [%]	Pseudo-SARS-CoV-2 [%]	Pseudo-SARS-CoV-2 + Ab [%]	MIX1 [%]	MIX2 [%]
CD38^-^CD24^+^	82.33 ± 0.45	82.47 ± 0.93	82.00 ± 0.44	**95.03 ± 0.38**	82.30 ± 0.96
Transitional	0.93 ± 0.06	**1.10 ± 0.10**	**1.33 ± 0.06**	**0.03 ± 0.06**	1.00 ± 0.35
CD38^+^24^-^	0.17 ± 0.06	0.20 ± 0.00	0.17 ± 0.06	**0.00 ± 0.00**	0.17 ± 0.06
CS	6.70 ± 0.20	**5.97 ± 0.06**	7.73 ± 0.74	**17.73 ± 2.61**	**5.77 ± 0.06**
NCS	14.97 ± 0.72	15.53 ± 1.42	**11.13 ± 0.15**	**12.57 ± 1.70**	**16.17 ± 0.64**
NAUVE	54.83 ± 0.55	55.00 ± 0.82	54.97 ± 1.16	**45.17 ± 2.25**	54.83 ± 1.10

PBMCs were treated with the following immunogenic factors: MOCK – LPS-treated cells (1.25 :g/ml), Pseud-SARS-CoV-2 – GeneCopoeia’s Lentifect™ SARS-CoV-2 Spike-pseudotyped lentivirus (100 VP/ml), Pseudo-SARS-CoV-2 + Ab – SARS-CoV-2 Spike Antibody (GeneCopoeia; 100 VP/ml + 2.62 μg/ml), MIX1 – AdV5/3-d24-ICOSL-CD40L + rRBD + pseudo-SARS-CoV-2 (100 VP/ml + 2.62 μg/ml+100 VP/ml), MIX2 – AdV5/3-d24-WT + rRBD+ pseudo-SARS-CoV-2 (100 VP/ml + 2.62 μg/ml + 100 VP/ml), CS – class switched subpopulation of CD38^-^CD24^+^, NCS – non-class switched subpopulation of CD38^-^CD24^+^, NAÏVE – cells unexposed to immunogenic factors; data are representative of three independent experiments; bolded number means *P*-value ≤ 0.05

### B cell class-switching induced by MIX1 (adenovirus adjuvant, rRBD, and pseudo-SARS-CoV-2)

**Fig. 2:**
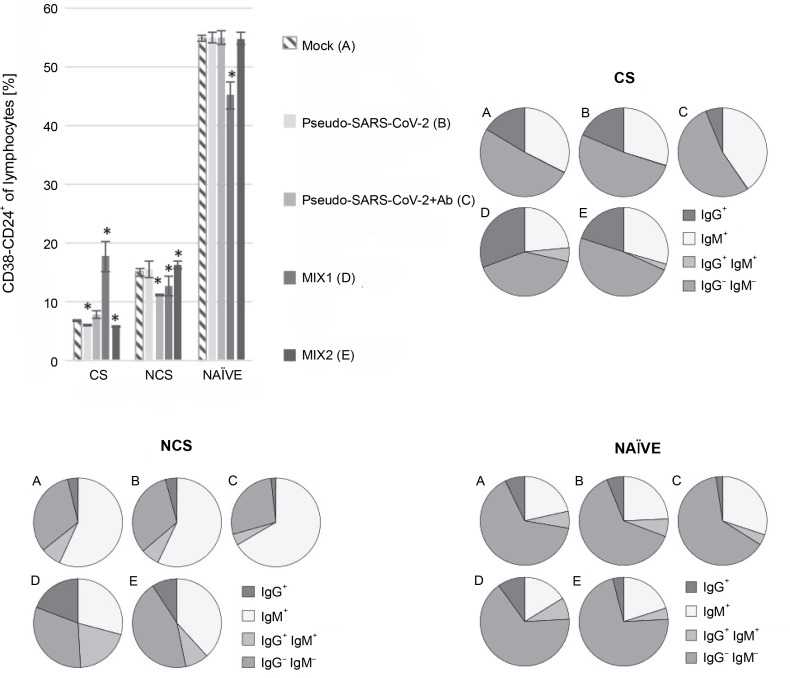
Percentage of the CD38^-^CD24^+^ cell subpopulations measured by flow cytometry (FACS Lyric, BD Biosciences, NJ, USA); PBMCs were treated for 24 h *in vitro* with the following immunogenic factors: A) Mock – LPS-treated cells (1.25 μg/ml), B) Pseudo-SARS-CoV-2 – GeneCopoeia’s Lentifect™ SARS-CoV-2 spike-pseudotyped lentivirus (100 VP/ml), C) Pseudo-SARS-CoV-2 +Ab – pseudo-SARS-CoV-2 + SARS-CoV-2 spike antibody (GeneCopoeia; 100 VP/ml + 2.62 μg/ml), D) MIX1 – AdV5/3-d24-ICOSL-CD40L + rRBD+ pseudo-SARS-CoV-2 (100 VP/ml + 2.62 μg/ml + 100 VP/ml), E) MIX2 – AdV5/3-d24-WT + rRBD+ pseudo-SARS-CoV-2 (100 VP/ml + 2.62 μg/ml + 100 VP/ml), CS – class-switched subpopulation of CD38^-^CD24^+^, NCS – non-class switched subpopulation of CD38^-^CD24^+^, and NAÏVE means cells unexposed to immunogenic factors; the data are representative of three independent experiments,*P-value ≤ 0.05

Regarding the subpopulation of MIX1-immunized CD38^-^CD24^+^ class-switched cells, significant differences were documented compared to the mock (Fig. 2). The CD38^-^CD24^+^IgG^+^ subsets showed a significant increase in their percentage after exposure to MIX1. The subset increase was as follows: ~1.9-fold for class-switched, ~5-fold for nonclass-switched, and ~1.4-fold for NAÏVE cells. The most negligible differences between treatments were found in the NAÏVE cell subpopulations. We observed a ~50-fold increase in IgG^+^IgM^+^ class-switched cells, a ~2.7-fold increase in IgG^+^IgM^+^ nonclass-switched cells, and a ~1.3-fold increase in IgG^+^IgM^+^ NAÏVE cells. To sum up, MIX1 induced an antigen-specific humoral response.

### T cell response to the immunogenic factors

**Fig. 3:**
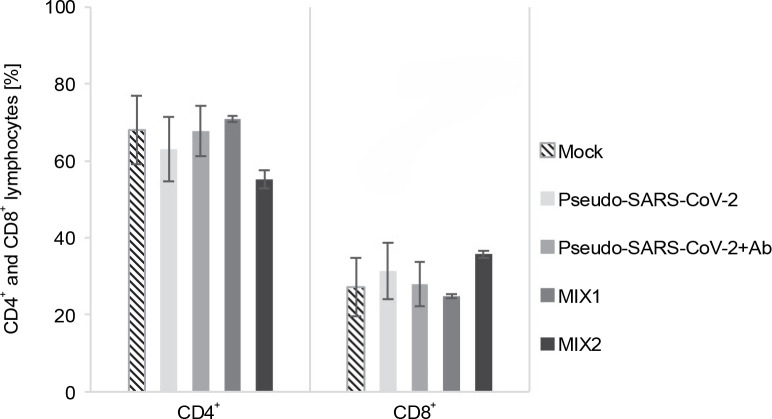
Immunogenic factor effects on lymphocytes T activation: MOCK – LPS-treated cells (1.25 μg/ml), Pseudo-SARS-CoV-2 – GeneCopoeia’s Lentifect™ SARS-CoV-2 spike-pseudotyped lentivirus (100 VP/ml), Pseudo-SARS-CoV-2 +Ab – pseudo-SARS-CoV-2 + SARS-CoV-2 spike antibody (GeneCopoeia; 100 VP/ml + 2.62 μg/ml), MIX1 – AdV5/3-d24-ICOSL-CD40L + rRBD+ pseudo-SARS-CoV-2 (100 VP/ml + 2.62 μg/ml + 100 VP/ml), MIX2 – AdV5/3-d24-WT + rRBD + pseudo-SARS-CoV-2 (100 VP/ml + 2.62 μg/ml + 100 VP/ml); the data are representative of 2–4 independent experiments; *P*-value > 0.05

**Fig. 4:**
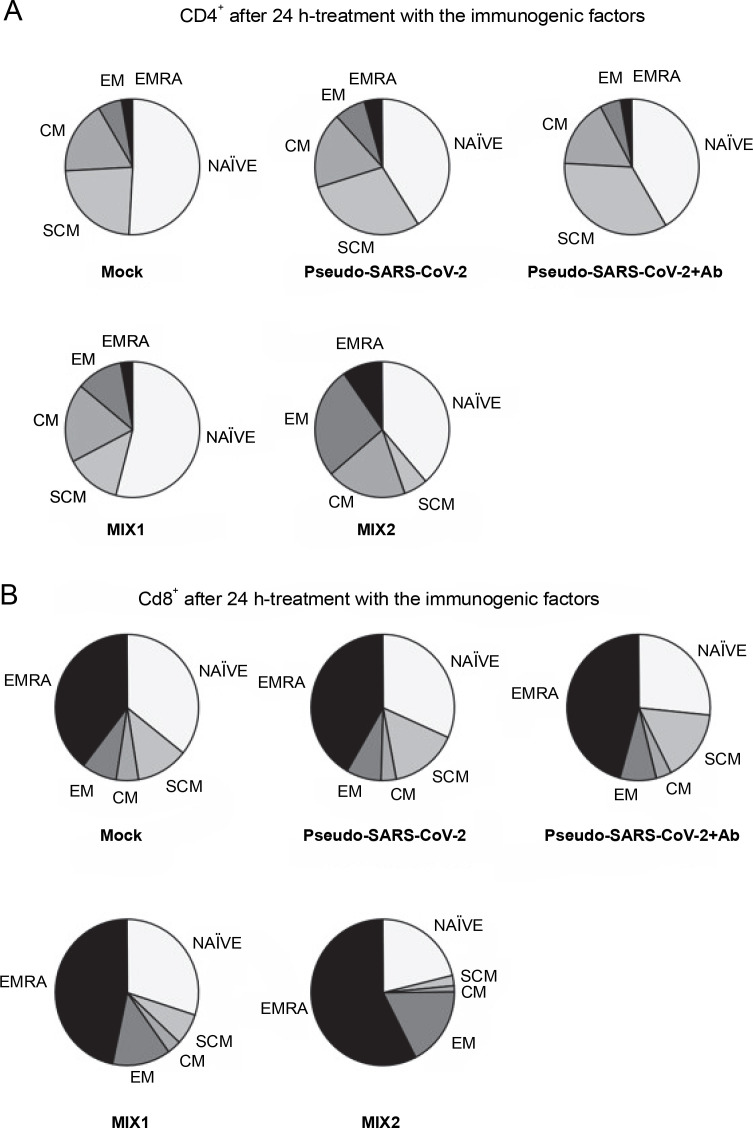
CD4^+^ (A) and CD8^+^ (B) cell subpopulations were measured by flow cytometry (FACS Lyric, BD Biosciences, NJ, USA); PBMCs were treated for 24 h *in vitro* with the following immunogenic factors: MOCK – LPS-treated cells (1.25 μg/ml), Pseudo-SARS-CoV-2 – GeneCopoeia’s Lentifect™ SARS-CoV-2 spike-pseudotyped lentivirus (100 VP/ml), Pseudo-SARS-CoV-2 +Ab – pseudo-SARS-CoV-2 + SARS-CoV-2 spike antibody (GeneCopoeia; 100 VP/ml + 2.62 μg/ml), MIX1 – AdV5/3-d24-ICOSL-CD40L + rRBD+ pseudo-SARS-CoV-2 (100 VP/ml + 2.62 μg/ml + 100 VP/ml), MIX2 – AdV5/3-d24-WT + rRBD + pseudo-SARS-CoV-2 (100 VP/ml + 2.62 μg/ml + 100 VP/ml); EMRA means terminally differentiated effector memory cells reexpressing CD45RA; NAÏVE means cells unexposed to immunogenic factors; SCM means stem-cell-like cells; CM means central memory cells; EM means effective memory cells; the data are representative of 2–4 independent experiments

**Fig. 5:**
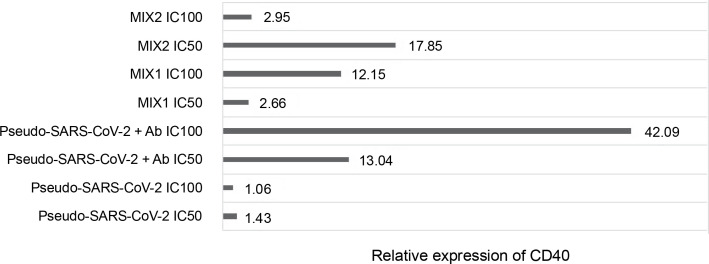
Fold gene expression of the CD40 receptor in VERO E6 cell line measured by RT-qPCR (BioRad); VERO E6 cells were treated for 24h *in vitro* with the following immunogenic factors: Pseudo-SARS-CoV-2 IC_100_/IC_50_ – GeneCopoeia’s Lentifect™ SARS-CoV-2 spike-pseudotyped lentivirus (100 VP/ml; 50 VP/ml), Pseudo-SARS-CoV-2+Ab – GeneCopoeia’s Lentifect™ SARS-CoV-2 spike-pseudotyped lentivirus + SARS-CoV-2 spike antibody (GeneCopoeia; 100 VP/ml + 5.24 μg/ml; 50 VP/ml + 2.62 μg/ml), MIX1 IC_100_/IC_50_ – AdV5/3-d24-ICOSL-CD40L + rRBD+ GeneCopoeia’s Lentifect™ SARS-CoV-2 Spike-pseudotyped lentivirus (100 VP/ml + 5.24 μg/ml + 100 VP/ml; 50 VP/ml + 2.62 μg/ml + 50 VP/ml), MIX2 – AdV5/3-d24-WT+ rRBD+ GeneCopoeia’s Lentifect™ SARS-CoV-2 Spike-pseudotyped lentivirus (100 VP/ml + 5.24 μg/ml + 100 VP/ml; 50 VP/ml + 2.62 μg/ml + 50 VP/ml)

The T cell subset changes in pooled lymphocytes were analyzed using flow cytometry (Fig. 3 and Fig. 4, Fig. S2 and Fig. S3, and Table S2 and Table S3). The response of PBMCs after 24-h treatment produced a more divergent phenotype in the CD8^+^ T cells compared to CD4^+^ T cells (Fig. 4 and Fig. 5). As shown in Table S4 and Figure S4, we observed a decrease in the number of NAÏVE cells when PBMCs were treated with pseudo-SARS-CoV-2 (40.47% ± 5.30 SD), pseudo-SARS-CoV-2+Ab (41.20% ± 10.74 SD), and MIX2 (38.73% ± 7.73 SD) compared to the mock (50.30% ± 6.35 SD).

The major decrease in the CD8^+^ T_SCM_ cells occurred in the samples treated with MIX1 (~1.7-fold) and MIX2 (~4-fold) compared to the mock. Conversely, we observed an increase in the number of CD8+ T_SCM_ cells when PBMCs were treated with pseudo-SARS-CoV-2 and pseudo-SARS-CoV-2+Ab. A significant increase was observed in the CD8^+^ T_EM_ population after treatment with MIX1 (~2-fold) and MIX2 (~4.7-fold). Of note, the CD8^+^ T_EMRA_ population increased when PBMCs were treated with MIX2 (9.63% ± 0.90 SD) compared to the mock (2.63% ± 1.96 SD).

We found that the CD8^+^ T_SCM_ cell populations decreased markedly when PBMCs were treated with MIX1 (6.83% ± 0.99 SD) and MIX2 (2.27% ± 1.98 SD) compared to the mock (10.63% ± 5.03 SD, *P* ≤ 0.05). Conversely, the CD8^+^ T_SCM_ cell number increased with both pseudo-SARS-CoV-2 (14.33% ± 8.06 SD) and pseudo-SARS-CoV-2+Ab (14.93% ± 7.13 SD). Notably, CD8^+^ T_EM_ cells were detected at about 12.87%±3.68 SD of the total CD4^+^ T cells when exposed to MIX1, and about 17.53%± 6.27 SD when exposed to MIX2. A significant increase in the number of CD8^+^ T_EMRA_ cells was observed when PBMCs were treated with pseudo-SARS-CoV-2+Ab (43.07% ± 5.72 SD), MIX1 (46.00% ± 5.76 SD), and MIX2 (57.57% ± 3.16 SD) compared to the mock (36.03% ± 3.20 SD, *P* ≤ 0.05).

### Quantification of the CD40 gene after treatment with the immunogenic factors

The relative CD40 expression was assessed using the RT-qPCR method (Fig. 5). The cells were treated with the immunogenic factors at IC_50_ (50 VP/ml and rRBD and/or antibody at 2.62 μg/ml) or IC_100_ (100 VP/ml and rRBD and/or antibody at 5.24 μg/ml) for 24 h *in vitro*. CD40 showed significant expression (*P* ≤ 0.05) in Vero E6 cells after treatment with pseudo-SARS-CoV-2+Ab (IC_100_), with a 42-fold upregulation compared to the untreated Vero E6 cells.

### Pseudo-SARS-CoV-2-based neutralization by antispike antibody

**Fig. 6:**
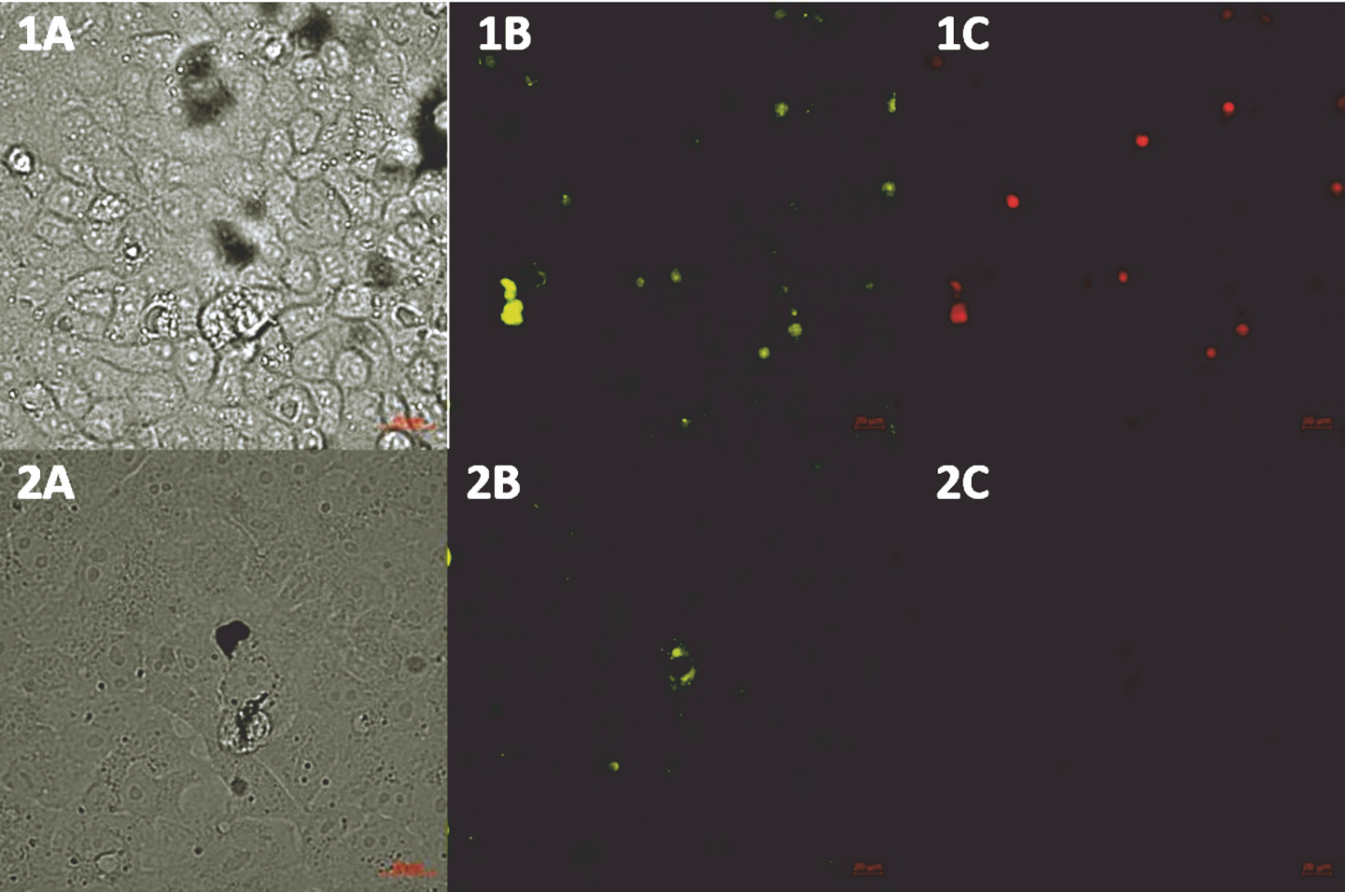
CSLM images (Zeiss, Germany) of SARS-CoV-2 Spike-pseudotyped lentivirus (10^-6^ RFU/ml, GeneCopoeia’s Lentifect™) – based neutralization assay in the Vero E6 cells expressing the human angiotensin-converting enzyme-2 (hACE2) receptor for SARS-CoV-2; A) transmitted light image, B) green fluorescence protein (eGFP) expressed by pseudo-SARS-CoV-2 bound by the Vero E6 receptors, C) propidium iodide (PI) fluorescence staining of the nucleus, showing the pattern of live and dead Vero E6 cells; 1A–1C) VERO E6 cell monolayer incubated with pseudo-SARS-CoV-2; 2A–C) VERO E6 cell monolayer incubated with pseudo-SARS-CoV-2 + SARS-CoV-2 spike antibody (GeneCopoeia; > 1000 RFU/ml + 5.24 μg/ml)

We performed a BSL-2 pseudovirus-based neutralization assay in Vero E6 cells expressing the human angiotensin-converting enzyme-2 (hACE2) receptor for SARS-CoV-2. We demonstrated that the SARS-CoV-2 spike is a useful tool for measuring the neutralization ability of candidate vaccines in preclinical models. As shown in Figure 6, a reduction in the intensity of eGFP was observed in the samples treated with the pseudo-SARS-CoV-2+Ab cocktail.

## Discussion

The protective immunity generated during the vaccination process is characterized by antigen-specific memory cells secreting effector molecules upon later infection. It includes all components of the host immune system, such as CD4^+^ T cells, CD8^+^ T cells, NK cells, and B cells. We used an *in vitro* immune response model to assess the immunogenicity of a composition containing a protein antigen (rRBD), adenoviral adjuvants (AdV5/3-D24-WT and AdV5/3-D24-ICOSL-CD40L), and GeneCopoeia’s Lentifect™ SARS-CoV-2 Spike-pseudotyped lentivirus.

We employed prime-boost immunization with rRBD and/or pseudo-SARS-CoV-2 spike to induce specific memory B cells (Fig. 2). The emergence of CD19^+^ cells (Fig. 1) was dependent on the adjuvant armed with ICOS and CD40 ligands (AdV1). Class-switched IgG^+^ and IgM^+^IgG^+^ B cells were noted when treated with AdV1 or spike-neutralizing antibody (Fig. 2). Generally, class-switched memory B cells responded to rRBD and/or pseudo-SARS-CoV-2 spike. As described by McHeyzer-Williams et al. (2011), the vaccine boost requires an adjuvant to induce class-switched memory B cells as the dominant precursor at recall. Our formula favors B cells and promotes switched antibodies for effective antigen-specific immunity (McHeyzer-Williams et al., 2011; Wang et al., 2021). Furthermore, we emphasized that memory B cells responding to rRBD and/or pseudo-SARS-CoV-2 spike require help from T cells (Fig. 3–5).

Engagement of the CD40 receptor is crucial in the initiation and progression of cellular and humoral adaptive immunity (McHeyzer-Williams et al., 2011). Including CD40L in a COVID-19 DNA vaccine reduced lung pathology more effectively than without the ligand (Tamming et al., 2021). Studies on convalescent macaques showed that targeting RBD to CD40 induces significant levels of specific T and B cells with long-term memory phenotypes (Marlin et al., 2021; Tamming et al., 2022). Treatment with pseudo-SARS-CoV-2+Ab (IC_100_ = 100 VP/ml) resulted in a significant (~42-fold) expression of CD40. Conversely, a slight change in CD40 expression was noted when VERO E6 cells were treated with pseudo-SARS-CoV-2 alone. The importance of CD40 ligand–receptor interactions in immune regulation and homeostasis is well-established (Grifoni et al., 2020).

In this study, we examined changes in the levels of CD19^+^CD24^+^CD38^+^ and CD19^+^CD24^+^CD38^-^ cells treated with immunogenic factors. In the FACS flow cytometry assay, we found that the CD19^+^CD24^+^CD38^-^ cell subset was upregulated under treatment with AdV1 armed with CD40L compared to the mock (Table 1, *P* ≤ 0.05). The class-switched B cells were induced in response to CD40L (Table 1 and Fig. 2). These cells are central to antibody-based immunological protection (McHeyzer-Williams et al., 2011). According to McHeyzer-Williams et al. (2011), modifying class-switched B cells provides a possibility for inducing antibody repertoires toward enhanced antigen binding.

Generally, SARS-CoV-2 is a new virus, and we know relatively little about its mechanisms of action and interaction with the host immune system, including CD4^+^ lymphocytes. Many studies have shown the responses of T cells from COVID-19 patients to different SARS-CoV-2-derived peptides matching sequences of S and N proteins. Furthermore, the expression of several cytokines like IFN-γ, IL-2, and IL-17 in CD4^+^ cells has been reported (Braun et al., 2020; Mateus et al., 2020; Meckiff et al., 2020; Sałkowska et al., 2020). A study by Weiskopf et al. showed that SARS-CoV-2 proteins S and N induce IFNG expression in CD4^+^ cells differentiated towards Th1 cells, while Th17 cells from the same individuals did not exhibit this response (Weiskopf et al., 2020).

We found that the spike leads to the activation of CD8^+^ T and CD4^+^ T cells, which are key players in antiviral responses and are advantageous for vaccines (Grifoni et al., 2020). We showed that the CD8^+^ TEMRA and TEM cells were differentiated in response to MIX1 (AdV5/3-d24-ICOSL-CD40L+rRBD+GeneCopoeia’s Lentifect™ SARS-CoV-2 Spike-pseudotyped lentivirus). In line with Melgaço et al. (2020), we reported an increased frequency of CD8^+^ T_CM_, T_EM_, and T_EMRA_ phenotypes in COVID-19 (primo-infected) individuals stimulated *in vitro* with the spike protein. Thus, MIX1 is effective in promoting an immune response similar to that acquired through infection, which is important for immune memory cell formation. Sharma and Rudra (2018) showed that CD8^+^ T_EMRA_ cells regulate cellular homeostasis in response to stress generated by immune factors. Moreover, we showed that CD4^+^ T_EM_ was activated after stimulation with MIX1. Our data corroborate the findings described by Grifoni et al. (2020) that central memory CD4^+^ T-cells are activated after stimulation with protein.

We indicated a correlation between the protection offered by vaccine regimens and their ability to induce high frequencies of multifunctional T cells. Seder et al. (2008) reviewed that viral adjuvants are used as a boost following priming with proteins for optimizing the CD8^+^ T-cell responses. In line with this, our vector was capable of eliciting potent T-cell responses. We showed that the specific SARS-CoV-2 Spike Antibody limits the internalization of pseudo-SARS-CoV-2. This result aligns with previous studies wherein antibody-mediated blocking of the receptor binding domain resulted in the inhibition of infection (Zhu, 2004).

## Conclusion

We proposed that targeting class-switched memory B cells with CD40L and rRBD and/or SARS-CoV-2 Spikepseudotyped lentivirus enhances immunological protection against COVID-19. The class-switched memory B cells were the dominant antigen-specific cells in primary reactions. Therefore, it is expected that the proposed formula will promote T-cell recognition upon subsequent exposure to viral particles or the whole virus.

## Supplementary Material

Induction of an immune response by a nonreplicating adenoviruses-based formulation versus a commercial pseudo-SARS-CoV-2 vaccine
