# Supplementary material for: Induction of an immune response by a nonreplicating adenoviruses-based formulation versus a commercial pseudo-SARS-CoV-2 vaccine
Source: BioTechnologia (Pozn). 2024 Sep 30;105(3):263–72. doi: 10.5114/bta.2024.141805 (PMC11492889; doi:10.5114/bta.2024.141805)
Supplement: Induction of an immune response by a nonreplicating adenoviruses-based formulation versus a commercial pseudo-SARS-CoV-2 vaccine [file BTA-105-3-54526-s001.pdf]

BioTechnologia vol. 105 (3) • 2024
Journal of Biotechnology, Computational Biology and Bionanotechnology SUPPLEMENTARY MATERIALS
https://doi.org/10.5114/bta.2024.141805
CC BY 4.0

Induction of an immune response by a nonreplicating adenoviruses-based formulation versus a commercial pseudo-SARS-CoV-2 vaccine

JOANNA BARAN [1], ŁUKASZ KURYK [2], MARIANGELA GAROFALO [3], KATARZYNA PANCER [2], MAGDALENA WIECZOREK [2], MICHALINA KAZEK [1*], MONIKA STANISZEWSKA [1*]

[1] Centre for Advanced Materials and Technologies, Warsaw University of Technology, Warszawa, Poland

[2] National Institute of Public Health National Institute of Hygiene (NIH) – National Institute of Research, Warszawa, Poland

[3] Department of Pharmaceutical and Pharmacological Sciences, University of Padova, Padova, Italy

Received: 8 April 2024; revised: 17 May 2024; accepted: 4 June 2024

Abstract

Screening for effective vaccines requires broad studies on their immunogenicity *in vitro* and *ex vivo*. We used a PBMC-based system to assess changes in $CD4^+$ T cells, $CD8^+$ T cells, and $CD19^+$ B cells upon stimulation with different combinations of antigens and adjuvants. We studied the activation mechanism using flow cytometry and two different adenoviral adjuvants characterized by the presence or absence of costimulatory ligands for the ICOS and CD40 receptors. Our studies identified the cellular targets and molecular mechanisms driving ongoing switched-antibody diversification. Class-switched memory B cells were the main precursor cells (95.03% ± 0.38 vs. mock 82.33% ± 0.45, $P < 0.05$) after treatment with the immunogenic formula: adenovirus armed (MIX1) or not (MIX2) with the ICOS and CD40 ligand, the recombinant receptor binding domain (rRBD), and Lentifect™ SARS-CoV-2 spike-pseudotyped lentivirus (GeneCopoeia, USA). Bcell class-switching towards the IgG^+IgM^+-positive phenotypes was noted (~50-fold increase vs. mock, $P < 0.05$). A significant increase was observed in the $CD8^+T_{EM}$ population of the MIX1 (~2-fold, $P < 0.05$) and MIX2 (~4.7-fold, $P < 0.05$) treated samples. $CD8^+T_{EMRA}$ increased when PBMCs were treated with MIX2 (9.63% ± 0.90, $P < 0.05$) vs. mock (2.63% ± 1.96). Class-switched memory B cells were the dominant antigen-specific cells in primary reactions. We indicated a correlation between the protection offered by vaccine regimens and their ability to induce high frequencies of multifunctional T cells.

Key words: immune response, adenoviruses, vaccines, SARS-CoV-2, CD cells

* Corresponding authors: Centre for Advanced Materials and Technologies, Warsaw University of Technology, Warszawa, Poland; e-mail: michalina.kazek@pw.edu.pl; monika.staniszewska@pw.edu.pl

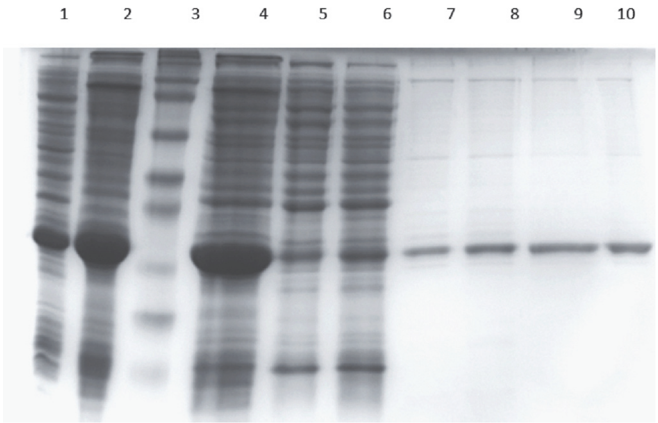

Fig. S1. SDS-PAGE analysis of rRBD expression in *E. coli* and purification on NiNTA Sepharose SuperFlow chromatography; proteins were separated in 15% acrylamide gel and stained by Coomassie Brilliant Blue; lanes: 1 – lysate of *E. coli* transformed with pT7/RBD plasmid, 2 – bacterial cell pellet after sonication, 3 – protein molecular marker (12.0–225.0 kDa) (Full-Range Rainbow, Amersham, UK), 4 – dissolved inclusion bodies (50 mM phosphate buffer, 5 mM β-mercaptoethanol, 7 M urea pH 12.0), 5 – protein unbound to NiNTA Sepharose column, 6 – fractions after washing (50 mM phosphate buffer pH 7.0, 7 M urea, 300 mM NaCl, 25 mM imidazole), 7, 8, 9 – fractions eluted from NiNTA Sepharose column (50 mM phosphate buffer pH 7.0, 7 M urea, 300 mM NaCl, 300 mM imidazole), and 10 – rRBD protein after elution and 24 h dialysis (50 mM phosphate buffer pH 8.0, 10% glycerol)

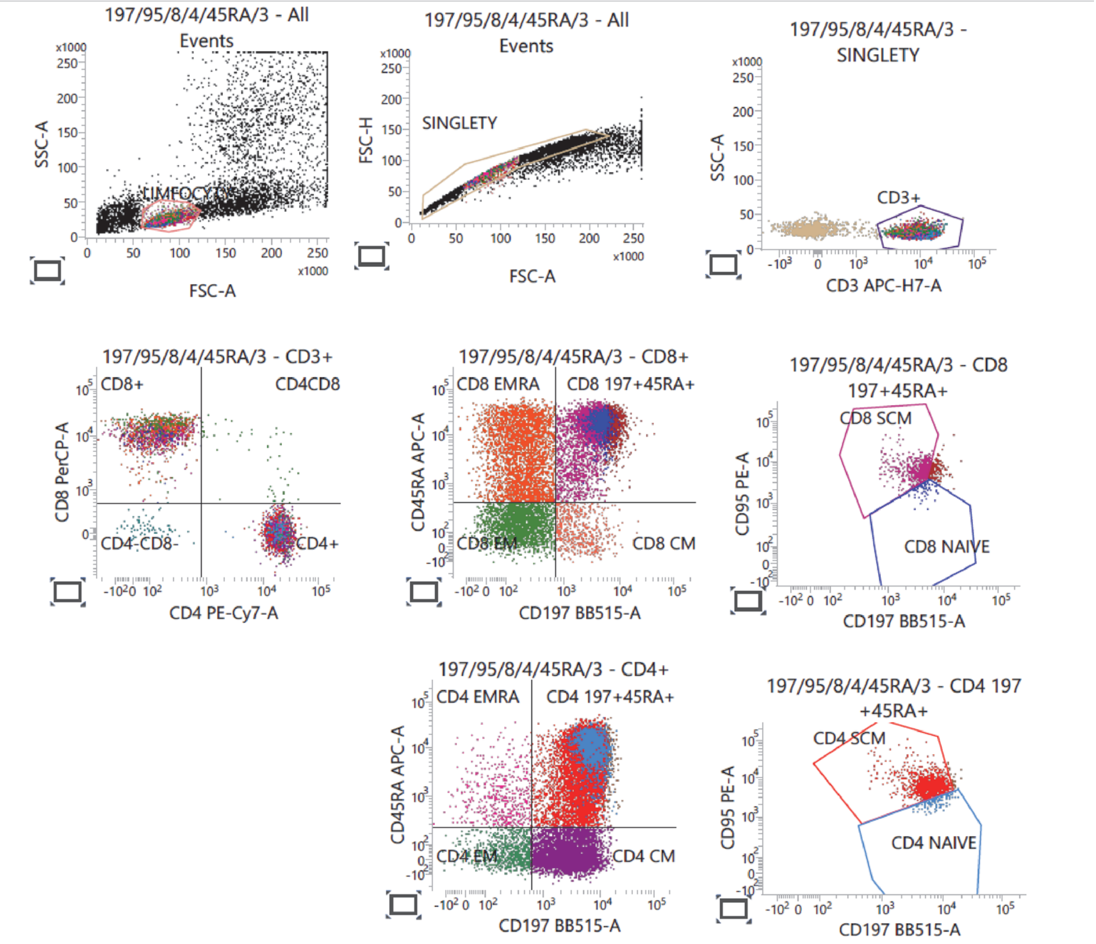

Fig. S2. Gating strategy of T-cell panel – CD197, CD95, CD8, CD4, CD45RA, and CD3

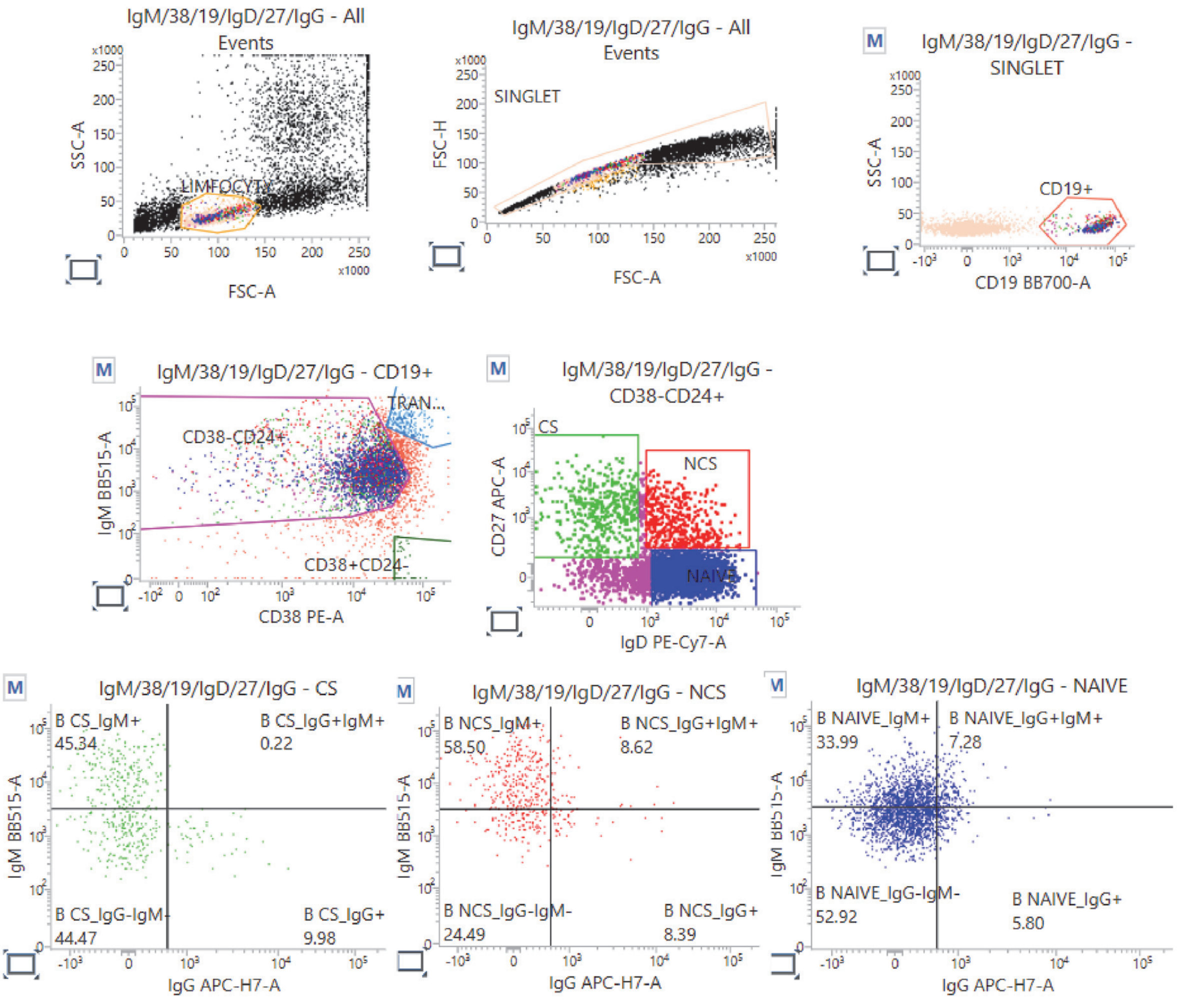

Fig. S3. Gating strategy of B-cell panel – IgM, CD38, CD19, IgD, CD27, and IgG

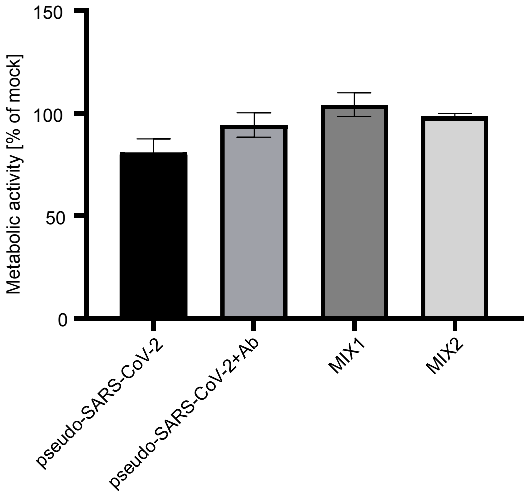

Fig. S4. Metabolic activity of PBMCs; results from MTS assay, representative figure; **Mock** – LPS-treated cells (1.25 μg/ml), **Pseudo-SARS-CoV-2** – GeneCopoeia's Lentifect™ SARS-CoV-2 – Spike-pseudotyped lentivirus (100 VP/ml), **Pseudo-SARS-CoV-2+Ab** – pseudo-SARS-CoV-2 + SARS-CoV-2 Spike Antibody (GeneCopoeia; 100 VP/ml + 2.62 μg/ml), **MIX1** – AdV5/3-d24-ICOSL-CD40L + rRBD + pseudo-SARS-CoV-2 (100 VP/ml + 2.62 μg/ml + 100 VP/ml), **MIX2** – AdV5/3-d24-WT + rRBD + pseudo--SARS-CoV-2 (100 VP/ml + 2.62 μg/ml + 100 VP/ml); *P*-value > 0.05

Table S1. Sequence of rRBD

aminoacid sequence

5′ Met + RBD + FT4 + SerGlySer + 6HisTag + STOP 3′ (232 aa = 696 bp)

MNITNLCPFGE VFNATRFASV YAWNRKRISN CVADYSVLYN SASFSTFKCY GVSPTKLNDL CFTNVYADSF VIRGDEVRQI APGQTGKIAD YNYKLPDDFT GCVIAWNSNN LDSKVGGNYN YLYRLFRKSN LKPFERDIST EIYQAGSTPC NGVEGFNCYF PLQSYGFQPT NGVGYQPYRV VVLSFELLHA PATV GYIPEAPRDG QAYVRKDGEW VLLSTFL SGSHHHHHH+ STOP

nucleotide sequence (codon usage *E. coli*)

5′ ATG AAC ATT ACC AAC CTG TGC CCG TTC GGC GAA GTG TTC AAC GCG ACC AGA TTC GCG TCT GTG TAT GCG TGG AAC CGT AAA CGT ATT TCT AAC TGC GTG GCG GAT TAT TCT GTG CTG TAT AAC TCT GCG TCT TTT TCT ACC TTT AAA TGC TAT GGC GTG TCT CCG ACC AAA CTG AAC GAT CTG TGC TTT ACC AAC GTG TAT GCG GAT TCT TTT GTG ATT CGT GGC GAT GAA GTG CGT CAG ATT GCG CCG GGC CAG ACC GGC AAA ATT GCG GAT TAT AAC TAT AAA CTG CCG GAT GAT TTT ACC GGC TGC GTG ATT GCG TGG AAC TCT AAC AAC CTG GAT TCT AAA GTG GGC GGC AAC TAT AAC TAT CTG TAT CGT CTG TTT CGT AAA TCT AAC CTG AAA CCG TTT GAA CGT GAT ATT TCT ACC GAA ATT TAT CAG GCG GGC TCT ACC CCG TGC AAC GGC GTG GAA GGC TTT AAC TGC TAT TTT CCG CTG CAG TCT TAT GGC TTT CAG CCG ACC AAC GGC GTG GGC TAT CAG CCG TAT CGT GTG GTG GTG CTG TCT TTT GAA CTG CTG CAT GCG CCG GCG ACC GTG GGC TAT ATT CCG GAA GCG CCG CGT GAT GGC CAG GCG TAT GTG CGT AAA GAT GGC GAA TGG GTG CTG CTG TCT ACC TTT CTG TCT GGT TCT CAT CAC CAT CAT CAC CAT TAA 3′

Source: https://www.ncbi.nlm.nih.gov/nuccore/1798174254/organism="Severe acute respiratory syndrome coronavirus2"/isolate="Wuhan-Hu-1"/host="Homo sapiens"/country="China"/collection_date="Dec-2019"

Table S2. Percentage of the $CD38^-CD24^+$ cell subpopulations measured by flow cytometry (FACS Lyric, BD Biosciences, NJ, USA)

B cell subsets		Immunogenic factors				
		Mock	Pseudo-SARS-CoV-2	Pseudo-SARS-CoV-2 + Ab	MIX1	MIX2
CS	IgM^+	32.50 ± 3.04	29.81 ± 1.55	**40.40 ± 2.46**	**23.50 ± 2.50**	29.49 ± 1.77
	IgG^+IgM^+	0.10 ± 0.14	0.11 ± 0.16	0.08 ± 0.12	**5.01 ± 2.00**	**2.24 ± 0.54**
	IgG^-IgM^-	51.04 ± 1.96	51.50 ± 1.30	53.29 ± 0.21	40.78 ± 7.36	48.25 ± 0.27
	IgG^+	16.37 ± 1.32	18.59 ± 2.71	**6.23 ± 2.38**	30.71 ± 7.83	**20.02 ± 1.72**
NCS	IgM^+	56.76 ± 1.47	56.99 ± 4.08	**66.17 ± 1.90**	**29.29 ± 10.02**	**38.25 ± 1.70**
	IgG^+IgM^+	7.49 ± 0.31	6.78 ± 0.68	**4.14 ± 0.64**	19.99 ± 8.70	8.55 ± 1.52
	IgG^-IgM^-	31.92 ± 1.68	32.02 ± 2.69	27.40 ± 1.90	31.75 ± 6.79	**44.01 ± 3.62**
	IgG^+	3.83 ± 0.38	4.21 ± 0.73	**1.80 ± 0.74**	19.31 ± 8.37	**9.19 ± 0.81**
NAÏVE	IgM^+	21.49 ± 2.01	24.28 ± 0.92	**30.11 ± 2.71**	16.17 ± 3.08	20.05 ± 1.16
	IgG^+IgM^+	6.18 ± 0.98	6.44 ± 0.44	**3.85 ± 0.72**	7.92 ± 2.54	**4.11 ± 0.25**
	IgG^-IgM^-	65.13 ± 3.41	63.14 ± 1.48	63.59 ± 1.64	65.98 ± 3.84	**71.85 ± 0.87**
	IgG^+	7.20 ± 0.74	6.14 ± 0.34	**2.44 ± 0.46**	9.93 ± 4.42	**3.98 ± 0.69**

PBMCs were treated for 24 h *in vitro* with the following immunogenic factors: **Mock** – LPS-treated cells (1.25 μg/ml), **Pseudo-SARS-CoV-2** – GeneCopoeia's Lentifect™ SARS-CoV-2 Spike-pseudotyped lentivirus (100 VP/ml), **Pseudo-SARS-CoV-2+Ab** – pseudo-SARS-CoV-2 + SARS-CoV-2 Spike Antibody (GeneCopoeia; 100 VP/ml + 2.62 μg/ml), **MIX1** – AdV5/3-d24-ICOSL-CD40L + rRBD + pseudo-SARS-CoV-2 (100 VP/ml + 2.62 μg/ml + 100 VP/ml), **MIX2** – AdV5/3-d24-WT + rRBD + pseudo-SARS-CoV-2 (100 VP/ml + 2.62 μg/ml + 100 VP/ml); **CS** means class switched subpopulation of $CD38^-CD24^+$, **NCS** means nonclass switched subpopulation of $CD38^-CD24^+$, **NAÏVE** means cells unexposed to immunogenic factors; the data are representative of 2–4 independent experiments; bolded numbers P-value ≤ 0.05

Table S3. Percentage of different $CD4^+$ subpopulations measured by flow cytometry (FACS Lyric, BD Biosciences, NJ, USA)

$CD4^+$ subpopulations	Immunogenic factor				
	Mock	Pseudo-SARS-CoV-2	Pseudo-SARS-CoV-2 +Ab	MIX1	MIX2
	[%]				
$CD197^+CD45RA^+$	74.23 ± 10.28	70.40 ± 8.08	75.93 ± 4.16	67.30 ± 6.85	**44.60 ± 9.62**
NAÏVE	50.30 ± 6.35	40.47 ± 5.30	41.20 ± 10.74	53.67 ± 7.42	38.73 ± 7.73
SCM	23.03 ± 5.03	28.67 ± 8.06	34.03 ± 7.13	**13.20 ± 0.99**	**5.70 ± 1.98**
CM	17.40 ± 4.52	17.97 ± 3.63	16.33 ± 2.46	18.73 ± 2.33	19.07 ± 0.47
EM	5.70 ± 3.89	7.40 ± 2.83	5.00 ± 1.56	11.10 ± 4.10	**26.70 ± 9.19**
EMRA	2.63 ± 1.96	4.23 ± 1.67	2.70 ± 1.70	2.83 ± 0.74	**9.63 ± 0.90**

PBMCs were treated for 24 h *in vitro* with the following immunogenic factors: **MOCK** – LPS-treated cells (1.25 μg/ml), **Pseudo-SARS-CoV-2** – GeneCopoeia's Lentifect™ SARS-CoV-2 Spike-pseudotyped lentivirus (100 VP/ml), **Pseudo-SARS-CoV-2+Ab** – pseudo-SARS-CoV-2 + SARS-CoV-2 Spike Antibody (GeneCopoeia; 100 VP/ml + 2.62 μg/ml), **MIX1** – AdV5/3-d24-ICOSL-CD40L + rRBD + pseudo-SARS-CoV-2 (100 VP/ml + 2.62 μg/ml + 100 VP/ml), **MIX2** – AdV5/3-d24-WT + rRBD + pseudo-SARS-CoV-2 (100 VP/ml + 2.62 μg/ml + 100 VP/ml); **CS** means class switched subpopulation of $CD38^-CD24^+$, **NCS** means nonclass switched subpopulation of $CD38^-CD24^+$, **NAÏVE** means cells unexposed to immunogenic factors; bolded number means P-value ≤ 0.05

Table S4. $CD8^+$ subpopulations were measured by flow cytometry (FACS Lyric, BD Biosciences, NJ, USA)

$CD8^+$ subpopulations	Immunogenic factor				
	Mock	Pseudo-SARS-CoV-2	Pseudo-SARS-CoV-2 +Ab	MIX1	MIX2
	[%]				
$CD197^+$ $CD45RA^+$	52.2 ± 4.54	50.07 ± 2.15	46.07 ± 7.95	**38.07 ± 7.29**	**23.53 ± 3.21**
NAÏVE	32.47 ± 6.35	29.9 ± 5.30	25.1 ± 10.74	29.57 ± 7.42	21.27 ± 7.73
SCM	10.63 ± 5.03	14.33 ± 8.06	14.93 ± 7.13	**6.83 ± 0.99**	**2.27 ± 1.98**
CM	4.53 ± 1.16	3.2 ± 0.83	3.23 ± 1.14	3.07 ± 1.14	**1.33 ± 0.05**
EM	7.23 ± 1.86	7.43 ± 1.06	7.67 ± 2.89	12.87 ± 3.68	17.53 ± 6.27
EMRA	36.03 ± 3.20	39.3 ± 2.34	43.07 ± 5.72	46.00 ± 5.76	**57.57 ± 3.16**

PBMCs were treated for 24 h *in vitro* with the following immunogenic factors: **MOCK** – LPS-treated cells (1.25 μg/ml), **Pseudo-SARS-CoV-2** – GeneCopoeia's Lentifect™ SARS-CoV-2–Spike-pseudotyped lentivirus (100 VP/ml), **Pseudo-SARS-CoV-2+Ab** – pseudo-SARS-CoV-2 + SARS-CoV-2 Spike Antibody (GeneCopoeia; 100 VP/ml + 2.62 μg/ml), **MIX1** – AdV5/3-d24-ICOSL-CD40L + rRBD + pseudo-SARS-CoV-2 (100 VP/ml + 2.62 μg/ml + 100 VP/ml), **MIX2** – AdV5/3-d24-WT + rRBD + pseudo-SARS-CoV-2 (100 VP/ml + 2.62 μg/ml + 100 VP/ml); **EMRA** means terminally differentiated effector memory cells re-expressing CD45RA; **NAÏVE** means cells unexposed to immunogenic factors; **SCM** means stem-cell-like cells; **CM** means central memory cells; **EM** means effective memory cells; bolded number means P-value ≤ 0.05
